# Electrophysiological Correlates of the Autobiographical Implicit Association Test (aIAT): Response Conflict and Conflict Resolution

**DOI:** 10.3389/fnhum.2016.00391

**Published:** 2016-08-30

**Authors:** Maddalena Marini, Sara Agosta, Giuseppe Sartori

**Affiliations:** ^1^Department of Neurobiology, Harvard Medical School, Harvard UniversityBoston, MA, USA; ^2^Department of Psychology, Harvard UniversityCambridge, MA, USA; ^3^Center for Neurosciences and Cognitive Systems@UniTN, Istituto Italiano di TecnologiaRovereto, Italy; ^4^Department of General Psychology, University of PadovaPadua, Italy

**Keywords:** autobiographical memory, aIAT, deception, N200, LPC

## Abstract

The autobiographical IAT (aIAT) is an implicit behavioral instrument that can detect autobiographical memories encoded in an individual's mind by measuring how quickly this person can categorize and associate sentences related to a specific event with the logical dimensions *true* and *false*. Faster categorization when an event (e.g., I went to Paris) is associated with the dimension *true* than *false* indicates that that specific event is encoded as true in the individual's mind. The aim of this study is to investigate the electrophysiological correlates of the aIAT, used as a memory-detection technique (i.e., to identify which of two events is true). To this end, we recorded ERPs while participants performed an aIAT assessing which of two playing cards they had previously selected. We found an increased N200 and a decreased LPC (or P300) at the fronto-central sites when participants associated the selected playing card with the dimension *false* than *true*. Notably, both components have been previously and consistently reported in studies investigating deception. These results suggest that associating a true autobiographical event with the concept of *false* may involve the same cognitive processes associated with deception.

## Introduction

Deception can be defined as a deliberative attempt of a person to create in another a belief which he/she considers to be untrue (Vrij, 2001).

Given its obvious relevance in several settings (e.g., in the forensic setting; Meijer et al., 2010), the study of deception has raised considerable interest among researchers and many studies sought to investigate its underlying cognitive processes by means of modern brain imaging techniques such as functional Magnetic Resonance Imaging (fMRI) and Event Related Potentials (ERPs; Langleben et al., 2002, 2005; Ganis et al., 2003, 2011; Johnson et al., 2003; Spence et al., 2004; Sokolovsky et al., 2011; Hu and Rosenfeld, 2012). For example, fMRI studies showed that the patterns of activations associated with deception involve predominantly frontal areas, such as the dorsolateral prefrontal cortex (DLPFC) and the anterior cingulate cortex (ACC), which also have a role in the inhibition and control of automatic responses (Sip et al., 2008). Consistently with these results, ERP studies (Rosenfeld et al., 1998; Tardif et al., 2000; Soskins et al., 2001; Miller et al., 2002; Johnson et al., 2003, 2008) showed that the recognition of deceptive behaviors elicits two electrophysiological components typically associated with conflicting responses: the N200 and the LPC (or also known as P300). The N200 is a negative-going component that occurs around 200–350 ms post-stimulus and is thought to be related to conflict detection (Van Veen and Carter, 2002; Folstein and Van Petten, 2008). The LPC is a positive wave that starts around 300 ms after stimulus onset and reflects increased cognitive load (Isreal et al., 1980a,b; Wickens et al., 1983; Kramer et al., 1985). For example, Hu et al. (2011) investigated deception by means of a Differentiation of Deception Paradigm (DDP; Furedy et al., 1988), in which participants answered the same questions about self- and other-related information twice, once truthfully and once deceptively. In this paradigm, honest and deceptive responses occurred in equal proportion for participants (i.e., 50–50%). The authors found that lying was associated with a decreased LPC (or also known as P300) and an increased N200 in the fronto-central area. Similarly Suchotzki et al. (2015), used a Sheffield Lie Test (a modified version of the DDP) in which participants were presented with the same set of questions (i.e., mock-crime and control questions): in half of the trials they were instructed to give honest responses while in the other half to give deceptive responses. They found a decreased LPC and an enhanced N200 over fronto-central electrodes for deceptive responses compared to honest responses.

Taken together, these findings suggest that lying elicits cognitive processes associated with the inhibition of an automatic response (i.e., the truth) and an increase in cognitive load needed to generate an alternative response (i.e., the deception; Spence et al., 2001).

We recently proposed a novel paradigm relying on reaction times (RTs) that might be used to investigate deception: the autobiographical IAT (aIAT; Sartori et al., 2008). The aIAT is a variant of the Implicit Association Test (Greenwald et al., 1998; Nosek et al., 2007) that assesses whether a specific autobiographical event is encoded as true or false in the respondent's memory by measuring how quickly a person can categorize and associate sentences related to an autobiographical event with the logical dimensions *true* and *false*. For example, in a typical aIAT assessing whether a given person has spent his/her last summer to Paris or to London, participants are asked to classify sentences representing the four categories—*Paris, London, true*, and *false*—by pressing one of two keys in two different response conditions. In one condition, participants categorize sentences related to *Paris* and to the dimension *true* with one response key, while categorizing sentences related to *London* and to the dimension *false* by using another response key. In the other condition, participants categorize the same sentences but with a different key configuration: this time one response key is used to categorize sentences related to *Paris* and to the dimension *false*, while the other response key is used to categorize sentences related to *London* and to the dimension *true*. The difference in average categorization latency between the two conditions is an indicator of association strengths between the autobiographical event (*Paris* or *London*) and the logical categories (*true* and *false*). For example, faster categorization when sentences related to Paris are associated with the dimension true (and sentences related to London to the dimension false) compared to the reverse indicates an implicit association of the logical dimension true with Paris compared to London. This result is usually interpreted as recognition of the event “I went to Paris” over “I went to London” as true.

The aIAT has demonstrated high accuracy and validity in several settings (Sartori et al., 2007, 2008; Marini et al., 2012; Agosta and Sartori, 2013), as well as its resistance to faking. Indeed, although studies showed that the aIAT can be faked when examinees are given specific instruction or previous training (Verschuere et al., 2009; Hu et al., 2012, 2015), recent research (Agosta et al., 2011) demonstrated that it is possible to identify successfully fakers on the basis of specific response patterns. Indeed, participants who try to fake the aIAT show different latencies in the test and practice blocks. That is, they are abnormally slow in the test blocks compared to the practice blocks of the aIAT.

The goal of the present study was to investigate the electrophysiological correlates of the aIAT used as a memory-detection technique. In particular, our working hypothesis was that the incongruent condition of the aIAT would elicit the same electrophysiological correlates associated with conflicting responses observed in studies investigating deception (i.e., LPC and N200).

Indeed, similar to lying, the incongruent condition of the aIAT, requires the inhibition of an automatic response (i.e., classify stimuli with a response configuration that is *congruent* with the true autobiographic event encoded in their memory: true event with the dimension true) and select a response which is in conflict with it (i.e., classify stimuli with a response configuration that is *incongruent* with the true autobiographic event encoded in their memory: true event with the dimension false).

In order to test our hypothesis, we recorded ERPs while participants performed an aIAT assessing which of two playing cards (e.g., 4 of diamonds and 7 of clubs) they had previously selected. We then compared the amplitudes of the LPC and N200 over the fronto-central electrodes recorded during the incongruent and congruent conditions.

## Methods

### Participants

Thirty-eight undergraduate students (29 women and 9 men; 19–31 years) in Psychology at University of Padua volunteered to take part in the experiment. All participants reviewed and signed an informed consent form in which the overall design of the study, risks, and the voluntary nature of participating in the study were explained. The study was approved by Ethics Committee of the Department of General Psychology of University of Padua.

### Materials and procedure

Before aIAT administration, participants were asked to select one of two covered playing cards, memorize it, and perform a consolidation task. In order to control the selected playing card, we randomly assigned participants to two groups. In the 4 of diamonds group, both covered playing cards were 4 of diamonds. In the 7 of clubs group, both covered playing cards were 7 of clubs. Of the 38 participants, 19 were assigned to *4 of diamonds* group and 19 to *7 of clubs* group.

In each trial of the consolidation task, one of eight different playing cards (e.g., 4 of diamonds, 7 of clubs, 3 of hearts, 3 of diamonds) was presented in the center of the screen. Participants were asked to press the space bar every time they saw the playing card that they previously selected. Each card was presented 5 times, for a total of 40 trials. Error feedback was presented for 400 ms if participants responded incorrectly.

In the aIAT, participants were asked to classify 10 sentences belonging to the logical dimensions true/false and 10 sentences referring to the 4 of diamonds or 7 of clubs playing cards (Table 1). Sentences belonging to the dimension true or false were composed of four words. Sentences referring to the 4 of diamonds or 7 of clubs were composed of three words followed by a picture representing one of the two playing cards.

**Table 1:** **List of sentences used in the experiment for the four categories**.

	**Italian**	**English translation**
**STIMULI**		
True	1. Ho davanti un computer. 2. Sto facendo un esperimento. 3. Sono in un laboratorio. 4. Sto usando una tastiera. 5. Mi trovo a Padova.	1. I am in front of a computer. 2. I am doing an experiment. 3. I am in a laboratory. 4. I am using a keyboard. 5. I am in Padua.
False	1. Ho davanti una televisione. 2. Sto facendo un gioco. 3. Sono in un cinema. 4. Sto usando una matita. 5. Mi trovo a Milano.	1. I am in front of a television. 2. I am doing a game. 3. I am in a cinema. 4. I am using a pencil. 5. I am in Milan.
4 of diamonds	1. Ho visto la carta (4 of diamonds picture). 2. Ho girato la carta (4 of diamonds picture). 3. Ho pescato la carta (4 of diamonds picture). 4. Ho selezionato la carta (4 of diamonds picture). 5. Ho preso la carta (4 of diamonds picture).	1. I saw the (4 of diamonds picture). 2. I turned the (4 of diamonds picture). 3. I picked the (4 of diamonds picture). 4. I cut the (4 of diamonds picture). 5. I took the (4 of diamonds picture).
7 of clubs	1. Ho visto la carta (7 of clubs picture). 2. Ho girato la carta (7 of clubs picture). 3. Ho pescato la carta (7 of clubs picture). 4. Ho selezionato la carta (7 of clubs picture). 5. Ho preso la carta (7 of clubs picture).	1. I saw the (7 of clubs picture). 2. I turned the (7 of clubs picture). 3. I picked the (7 of clubs picture). 4. I cut the (7 of clubs picture). 5. I took the (7 of clubs picture).

*The Table shows the Italian sentence and the corresponding English translation*.

**Figure 1:**
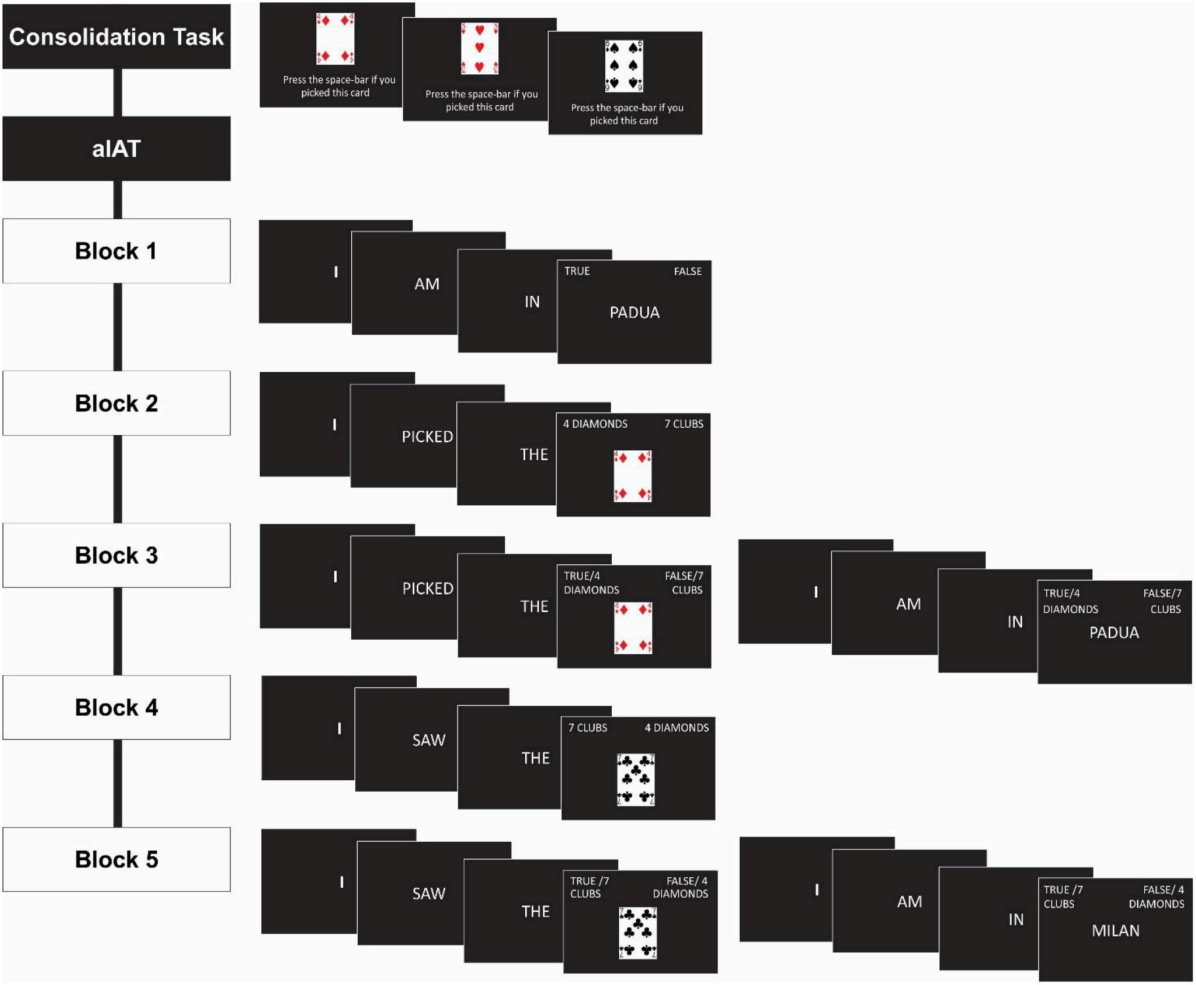
**Illustration of the experimental procedure in the consolidation task and aIAT**.

The aIAT consisted of a total of five blocks (Sartori et al., 2008) schematically shown in Figure 1. In Block 1 (20 trials; logical discrimination) participants classified sentences as true or false. They were asked to press the A key if the sentence was true for them (e.g., I am in a laboratory) and the L key if the factual sentence was false for them (e.g., I am in a theater).

In Block 2 (20 trials; playing card discrimination), participants categorized statements relative to 4 of diamonds or 7 of clubs. They pressed the A button to classify the sentences related to 4 of diamonds (e.g., I picked the 4 of diamond) and L button to classify the sentences referred to7 of clubs (e.g., I picked the 7 of clubs).

In Block 3 (60 trials; double categorization), participants categorized statements belonging either to the true/false dimension or 4 of diamonds/7 of clubs. They were asked to press the A key to classify sentences referring to 4 of diamonds and the dimension true, whereas the L key was used to classify sentences referring to the 7 of clubs and the dimension false. For participants in the 4 of diamonds group this was the *congruent* block, while for participants in the 7 of clubs group this was the *incongruent* block.

In Block 4 (40 trials; reversed playing card discrimination), participants classified only statements referring to 4 of diamonds or 7 of clubs as in Block 2 but using a different key configuration. They were asked to press the A key for the sentences related to 7 of clubs (7 of clubs category) and the L key for sentences related to 4 of diamonds (4 of diamonds category).

In Block 5 (60 trials; reversed double categorization), participants classified statements referring both the true/false dimension and 4 of diamonds/7 of clubs as in Block 3, but in this condition they pressed the A key for true statements and the sentences related to 7 of clubs, the L key for false statements and sentences related to 4 of diamonds^1^. Thus, for participants in the 4 of diamonds group this block was *incongruent*, while for participants in the 7 of clubs group this block was *congruent*.

Reminder labels in the form of category names (i.e., true, false, 4 of diamonds and 7 of clubs) remained on the monitor for the entire duration of each block. An error signal appeared after an incorrect response.

### EEG/ERP recording

Scalp voltages were recorded using a 64-channel electro-cap with Ag/AgCl electrodes. A frontal electrode (AFz) was connected to the ground. During recording, all electrodes were referenced to Cz. Vertical and horizontal eye movements were recorded. Electrode impedance was kept under 5 kΩ for all recordings. The EEG was recorded continuously and digitized at a sampling rate of 500 Hz. The signal was off- line filtered using a low-pass filter with cut-off frequency of 30 Hz and 24 dB/octave attenuation. Ocular movement artifacts were corrected using the algorithm provided by the Neuroscan 4.3 software. The EEG was segmented into epochs starting 100 ms before presentation of the target word and lasting 1500 ms after its onset. The epochs were aligned to the 200-ms baseline before onset of the target word presentation. Trials contaminated by movement artifacts (peak-to-peak deflection over ±75 μV) were rejected.

## Results

### Behavioral data

In the present experiment, we considered three dependent variables: D index (Greenwald et al., 2003), mean RTs and percent accuracy (PA) in the two combined categorization blocks (blocks 3 and 5). Responses faster than 150 ms or slower than 10,000 ms were removed. Following Greenwald et al. (2003), we computed the D index for each participant by dividing the difference in mean response latency between the two aIAT combined blocks by the participant's latency standard deviation inclusive of the two combined blocks. Errors were replaced with the mean of the correct responses in that response block plus a 600 ms of penalty (Greenwald et al., 2003). A positive D index indicated a stronger association of *4 of diamonds* card with *true* and *7 of clubs* card with *false*, whereas a negative D index indicated a stronger association of *4 of diamonds* card with *false* and *7 of clubs* card with *true*.

We analyzed participants' behavioral data by means of three analyses of variance (ANOVAs) on average RTs, PA, and D index. RTs and PA were submitted to ANOVA with congruency (congruent vs. incongruent) as a within subject factor and group (4 of diamonds and 7 of clubs) as a between subject factor. D index was submitted to an ANOVA with group (4 of diamonds and 7 of clubs) as a between subject factor.

In agreement with previous results (Sartori et al., 2008; Agosta and Sartori, 2013), faster RTs and higher PA were found in the congruent than in the incongruent condition [RTs: 621 vs. 773 ms, *F*_(1, 37)_ = 28.22, *p* < 0.01, $\eta_{\text{p}}^{2}=0.43$; PA: 0.97 vs. 0.94; *F*_(1, 37)_ = 16.66, *p* < 0.01, $\eta_{\text{p}}^{2}=0.31$]. Similarly, a significant difference in the D indexes was found between groups [*F*_(1, 36)_ = 46.98, *p* < 0.01, $\eta_{\text{p}}^{2}=0.57$]. That is, the D index was positive for the 4 of diamonds group and negative for the 7 of clubs group (0.55 vs. −0.37). This pattern of results indicated that the aIAT accurately detected which playing card participants had selected. Indeed, faster RTs were found when the *playing card that participants selected* was associated with the dimension *true* (congruent condition) and slower when it was instead associated with dimension *false* (incongruent condition). The D index accurately classified 34 out of 38 participants. Eight of these 34 participants showed *D* values ranging in the inconclusive window (−0.2, +0.2) identified by Agosta and Sartori (2013). The accuracy of the aIAT was confirmed by an ROC analysis (area under the curve, AUC = 0.93).

### Electrophysiological data

We next investigated the electrophysiological correlates of the aIAT by analyzing ERP responses. In this analysis, we excluded (1) participants who showed no stronger association between the selected card and the dimension true (i.e., participants for whom the D index calculated from aIAT performance did not detect accurately which playing card they had previously selected), (2) data of subjects who showed excessive movement artifacts or bad electrodes impendence (5% of the trials), and (3) trials of the aIAT in which participants were not accurate (4.5% of the trials). Finally, for each subject, we separately averaged the ERPs recorded during the congruent and incongruent blocks respectively. The data of 31 participants were included in our analysis.

**Figure 2:**
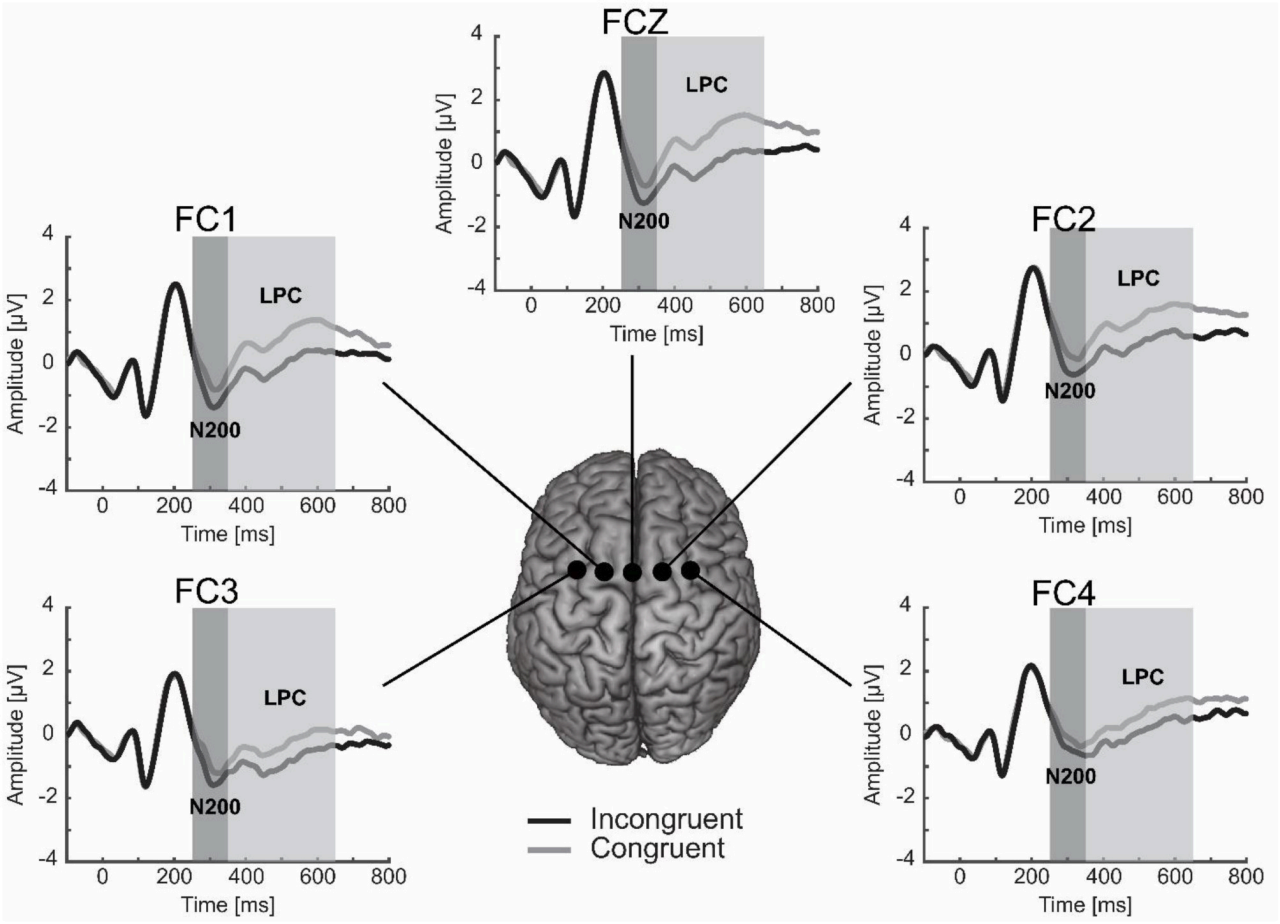
**The N200 and LPC in the fronto-central electrodes (FC3, FC1, FCZ, FC2, and FC4)**. The N200 was larger for the incongruent than congruent condition, while the LPC was smaller for the incongruent than congruent condition.

Visual inspection of the ERPs indicated two different components localized at the fronto-central electrodes (FC3, FC1, FCz, FC2, FC4): the N200 and the LPC (see Figure 2). Both components were measured from target stimulus onset both in the congruent and incongruent condition. The N200 was quantified as the average amplitude between 250 and 350 ms, whereas the LPC was determined as the mean voltage between 350 and 650 ms. To investigate the lateralization of these two ERP components, we calculated the mean amplitude of the fronto-central electrophysiological activity for electrodes covering the left (i.e., FC3 and FC1) and right (i.e., FC2 and FC4) anterior scalp regions for each participant and for each condition.

N200 and LPC mean amplitudes were separately submitted to an ANOVA with congruency (congruent vs. incongruent) and localization (left vs. center vs. right) as within subject factors. All *p*-values were corrected for violations of the sphericity assumption using the method of Greenhouse and Geisser (Greenhouse and Geisser, 1959; Picton et al., 2000).

#### N200

We found a significant main effect of congruency, *F*_(1, 30)_ = 6.52, *p* < 0.05, $\eta_{\text{p}}^{2}=0.18$, indicating a greater negativity for the incongruent than congruent condition (−1.60 vs. −1.13). Localization was also significant, *F*_(2, 60)_ = 10.28, *p* < 0.01, $\eta_{\text{p}}^{2}=0.25$. Bonferroni pairwise comparisons showed that the N200 amplitudes were more negative in the left and central electrodes compared to the right electrodes (−1.74 and −1.45 vs. −0.90, *p* < 0.01). The interaction between congruency and localization did not reach significance, *F*_(2, 60)_ = 0.51, *p* = 0.52, $\eta_{\text{p}}^{2}=0.02$, indicating that the difference between the N200 amplitudes in the congruent and incongruent conditions was not modulated by localization.

#### LPC

The main factor of congruency was significant, *F*_(1, 30)_ = 14.35, *p* < 0.01, $\eta_{\text{p}}^{2}=0.32$. More specifically the LPC amplitudes were smaller in the incongruent than congruent condition (0.26 vs. 0.86). We also found a significant effect of the localization factor, *F*_(2, 60)_ = 7.03, *p* < 0.01, $\eta_{\text{p}}^{2}=0.19$. A closer inspection revealed that the LPC amplitudes were more positive in the central and right electrodes compared to the left electrodes (0.73 and 0.85 vs. 0.10, *p* < 0.10). However, as in the case of the N200, the localization did not explain the difference between the LPC amplitudes in the congruent and incongruent conditions. Indeed, we found no significant effect of the interaction between congruency and localization factors, *F*_(2, 60)_ = 1.64, *p* = 0.21, $\eta_{\text{p}}^{2}=0.05$.

This pattern of results for the N200 and LPC was confirmed by a Spatial-Temporal Principal Component Analysis (ST-PCA; see Supplementary Materials).

Correlation analysis between the N200 and LPC amplitudes in the congruent and incongruent conditions by location showed that these two ERP components were positively correlated. That is, larger differences between the N200 amplitudes in the congruent and incongruent conditions were associated with larger differences in the LPC (left: 0.56, *p* < 0.001; center: *r* = 0.66, *p* < 0.001; right: *r* = 0.68, *p* < 0.001).

Taken together these results indicated that when participants associated the *playing card that they selected* with the dimension *false* (i.e., incongruent condition), both the N200 and LPC components elicited a negative pattern. Specifically, we observed an increase of the negativity of the N200 and a reduction of the positive deflection of the LPC in fronto-central electrodes.

## Discussion

The present study aimed at investigating the electrophysiological correlates of the aIAT used as a memory-detection technique. To this end, participants were first required to pick a card. They, then, underwent an aIAT in which we tested the strength of association between the card they had selected with the concept of true (congruent condition) or false (incongruent condition) while measuring ERPs from scalp electrodes. Our working hypothesis was that performance of the incongruent condition of the aIAT would elicit electrophysiological responses found in previous studies investigating deception: the N200 and the LPC. Consistently with our hypothesis we found a decreased LPC and an increased N200 in the incongruent condition of the aIAT.

### ERP results

In our experiments, the performance of the incongruent condition of the aIAT produced an increase of the N200 and a decrease of the LPC in the fronto-central region. Several studies support the association between these two components and deception.

Specifically, the LPC component has been consistently reported in many earlier studies and is considered a good indicator of deception (Rosenfeld et al., 1998; Tardif et al., 2000; Soskins et al., 2001; Miller et al., 2002; Johnson et al., 2003, 2008). For example, Johnson et al. (2003, 2008) reported that deceptive responses, conflicting with the truth, produced a reduced LPC amplitude. More recent studies, have found that deception elicits a more complex pattern of ERPs responses that involves not only the LPC but also the N200. In particular, studies using paradigms with an equal proportions of deceptive and honest responses showed, in addition to a reduced LPC, also a decreased N200 for lying than truth telling (Hu et al., 2011; Suchotzki et al., 2015). Interestingly, in the aIAT, similarly to paradigms used in more recent deception studies, participants had to associate, in different blocks, the same stimuli (i.e., sentences describing an autobiographical event) with the concepts of true and false. Taken together, our results show that, in agreement with our hypothesis, the incongruent block of the aIAT produces similar electrophysiological responses typically associated with deception.

The similarity between the electrophysiological responses associated with deceptive behaviors and the incongruent condition of the aIAT suggests that lying and associating a true event with the concept of false may reflect analogous cognitive processes. Indeed, during both deception and the incongruent block of the aIAT, the cognitive system needs to activate control processes in order (1) to inhibit a pre-potent response (i.e., to tell the truth in the case of deception and to associate a true autobiographical event with the concept of true in the case of the incongruent condition of the aIAT) and (2) to emit an alternative response (i.e., to lie in the case of deception and to associate a true autobiographical event with the concept of false in the case of the incongruent condition of the aIAT). The activation of such control processes is reflected in the pattern of ERPs responses that we reported here. Indeed, previous studies have consistently shown that the LPC and N200 are two ERP components reflecting a collection of cognitive control processes, such as response inhibition, detection of response conflict, and strategic performance monitoring (West, 2003; Folstein and Van Petten, 2008; Chen and Melara, 2009; Larson et al., 2009; Coderre et al., 2011; Wang et al., 2013). Specifically, a decreased LPC has been found in association with an increase of the cognitive load requested in a task (Magliero et al., 1984; Doucet and Stelmack, 1999). Similarly, an increase of the N200 over fronto-central scalp areas has been consistently reported in studies involving response inhibition (e.g., using the Go/Nogo task, Bokura et al., 2001; the Eriksen Flanker task, Bartholow et al., 2005; and the Stop Signal Paradigm, Schmajuk et al., 2006).

Note that whereas we and other previous studies found that the recognition of deception is associated with a decreased LPC, ERPs studies using the Concealed Information Test (CIT; Verschuere et al., 2011) showed an opposite result pattern. The CIT is a widely used instrument that, similar to the aIAT, allows to infer rather than detect deception. In the CIT, participants are presented with crime-relevant and irrelevant information. In particular, irrelevant information are presented with high probability while crime-relevant trials have a low probability of being presented (~10%). Our results, suggest that the CIT and the aIAT might engage different mechanisms. Indeed, in the CIT the recognition of crime-relevant information produces an increased LPC (or P300; Rosenfeld, 2011) while the incongruent block of the aIAT produces a decreased LPC. The different LPC patterns elicited by the aIAT and CIT might be ascribed to the nature of these two paradigms: the CIT is based on an oddball paradigm (Donchin and Coles, 1988) and thus reduces the probability of crime-relevant stimuli compared to irrelevant stimuli, while the aIAT uses the same proportion of stimuli in the congruent and incongruent conditions. The difference between these two instruments is further supported by a recent study showing no correlation between the two of them (Hu and Rosenfeld, 2012).

In a previous study from our group we showed that, when the aIAT is used as an intention-detection technique (i.e., to detect whether a specific prior intention is encoded as true in an individual's mind), the incongruent condition was associated with a reduced LPC (Agosta et al., 2011). Results reported here extend this result by showing that an additional component—i.e., the N200—is elicited by the incongruent condition when the aIAT is used to assess autobiographical memories. Taken together, these results suggest that the electrophysiological activations produced by the aIAT might differ on basis of the phenomenon assessed by this instrument.

### Behavioral results

Our study provided a further validation of the aIAT as a memory-detection technique at the behavioral level (AUC = 0.93). Indeed, the increase in reaction times in the incongruent condition of the aIAT is also in agreement with previous studies investigating deception suggesting that associating a true autobiographical event with the concept of false and lying has a cognitive cost. For example, Vrij and Mann (2001) found that the speech of a convicted murder was slower when lying that telling the truth. Similarly, studies investigating deception by means of RTs paradigms showed deceitful responses produced longer latencies in comparison with truthful responses (e.g., Spence et al., 2001, 2004; Carrión et al., 2010).

In addition to relevant results, the present study has also some limitations. It is important to point out that, while our data suggest a similarity in the cognitive processes associated with deception and the incongruent condition of the aIAT, the observation of similar electrophysiological brain activities is not enough to unambiguously support such conclusion. Indeed, we cannot conclusively exclude that additional cognitive processes may be responsible for the electrophysiological responses elicited by the aIAT in our study. A second limitation is that the autobiographical memory investigated in our study was emotionally neutral. Replication of our behavioral paradigm with other more “emotional-laden” memories (e.g., by using mock crime paradigm) or within more ecological settings will be particularly useful to increase the confidence of our results. Finally, although our results show that the aIAT can identify whether an event is associated with the concept of true than false, this does not necessary imply that the aIAT can detect whether an event is objectively true. Indeed, the aIAT reflects what is stored in an individual's mind. That is, if an event is strongly believed to be true and encoded as such in an individual's mind then the aIAT will identify it as a true event (Marini et al., 2012; Agosta and Sartori, 2013).

## Author contributions

Conceived the study: MM, SA, GS. Designed the study: MM, SA, GS. Performed the experiments: MM, SA. Analyzed the data: MM, SA. Interpretation of data: MM, SA, GS. Wrote the manuscript: MM, SA, GS.

### Conflict of interest statement

The authors declare that the research was conducted in the absence of any commercial or financial relationships that could be construed as a potential conflict of interest.

## Footnotes

^1^In each group, half of the participants administered the blocks in the order just outlined, whereas for the other half, the order of blocks 3 and 5 was reversed as well the order of blocks 2 and 4.
