# Supplementary material for: Electrophysiological Correlates of the Autobiographical Implicit Association Test (aIAT): Response Conflict and Conflict Resolution
Source: Front Hum Neurosci. 2016 Aug 30;10:391. doi: 10.3389/fnhum.2016.00391 (PMC5003893; doi:10.3389/fnhum.2016.00391)
Supplement: Supplementary file 1 [file supplementarymaterial.docx]

***Supplementary Material***

**Electrophysiological correlates of the autobiographical Implicit Association Test (aIAT): response conflict and conflict resolution**

**Maddalena Marini^*^, Sara Agosta and Giuseppe Sartori**

*** Correspondence:** Maddalena Marini, [Maddalena_Marini@hms.harvard.edu](mailto:Maddalena_Marini@hms.harvard.edu)

This file includes:

- Spatial-Temporal Principal Component Analysis and Results
- Figures S1 and S2
- Table S1
- References

**Spatial-Temporal Principal Component Analysis**

To further investigate the characteristics of the N200 and LPC components elicited during the performance of an aIAT we conducted a Spatial-Temporal Principal Component Analysis (ST-PCA) (Dien et al., 2003; Kayser and Tenke, 2006; Lui and Rosenfeld, 2008) on the ERPs component collected from 57 sites from stimulus onset to 650 ms (325 samples). We first performed a PCA on 20,150 patterns of spatial activation (31 subjects x 2 conditions x 325 time points) to identify a set of components that accounted most for the spatial variance in our data set. We retained components that explained more than 1.75% of the total variance [i.e., (1/number of all components extracted by the PCA)*100]. These components were then subjected to a varimax rotation(Dien, 2012). The scores associated with these components were then submitted to a temporal PCA. We retained temporal components that accounted for 95% of the total variance of our data and subjected them to a varimax rotation.

Scores obtained by spatial-temporal PCA were analyzed by a repeated-measures analysis of variance (ANOVA) with congruency (congruent and incongruent), spatial (1, 2, 3, 4, 5 and 6) and temporal (1,2,3,4,5 and 6) components as within-subjects factors. Significant interactions were analyzed by performing separate ANOVAs followed by Bonferroni-corrected pairwise comparisons.

**Results**

Spatial PCA. For the spatial PCA, we selected 6 components that accounted for 87.61% of the total variance: SC1=51.96%, SC2=15.28%, SC3=8.24%, SC4=5.77%, SC5=3.89% and SC6=2.50%. The spatial distribution of these components is shown in Figure S1.

Temporal PCA. The subsequent temporal PCA resulted in 7 components that accounted for 95.42% of the total variance: TC1=47.49%, TC2=23.14%, TC3=10.54%, TC4=6.81%, TC5=3.82%, TC6=2.16% and TC7=1.47%. Temporal components are presented in Figure S2.

ANOVA. Mauchly’s Test of Sphericity indicated a violation of the sphericity assumption, p<0.0005, and therefore we used a Greenhouse-Geisser correction for our analysis.

We found a significant three-way interaction between the factors congruency, spatial and temporal, F(3.72,11.65)=2.951, p<0.05, ɳ²p=0.090. Separate ANOVAs for each spatial component showed that the interaction between the factors congruency and spatial was significant only for the following components: SC2, F(1.72,51.65)=4.293, p<0.05, ɳ²p=0.125; SC3, F(1.91,57.23)=3.963, p<0.05, ɳ²p=0.117; SC4, F(1.53, 45.89)=4.512, p<0.05, ɳ²p=0.131 and SC6, F(1.82,54.55)=6.30, p<0.01, ɳ²p=0.173.

A closer inspection of these interactions showed that the factor congruency was significant only for a subset of the temporal components. That is, in the SC2 for the TC1, F(1,30)=8.687, p<0.01, ɳ²p=0.225, and TC5, F(1,30)=6.715, p<0.05, ɳ²p=0.183; in the SC3 for the TC4, F(1,30)=5.435, p<0.05, ɳ²p=0.153, and TC7, F(1,30)=7.630, p<0.01, ɳ²p=0.203; in the SC4 for the ST3, F(1,30)=5.147, p<0.05, ɳ²p=0.146, ST4, F(1,30)=5.052, p<0.05, ɳ²p=0.144, and ST7, F(1,30)=7.011, p<0.05, ɳ²p=0.189; and in the SC6 for ST1, F(1,30)=4.596, p<0.05, ɳ²p=0.133, ST4, F(1,30)=4.306, p<0.05, ɳ²p=0.126, and ST7, F(1,30)=10.225 p<0.01, ɳ²p=0.254.

It is worth emphasizing that the SC2 includes electrodes (i.e., FC1, FCZ and FC3) that were selected for the analysis presented in the main manuscript. Furthermore, TC1 and TC5 strongly overlapped with the temporal ranges (i.e., TC1=350-600 ms and TC5=250-450 ms) that we considered in our analysis of the LPC and N200 respectively. Most importantly, as our results showed, in the SC2 the two components TC1 and TC5 significantly differed between congruent and incongruent condition. This pattern of results matches the findings presented in the main manuscript, which were obtained by a hypothesis-driven selection of the electrodes and time ranges of interest.

The ANOVA revealed other effects that we report without further discussion below, because they are not relevant to the hypothesis under test.

We found a significant effect of the factors congruency, F(1,30)=4.292, p<0.05, ɳ²p=0.125, and spatial, F(2.42,72.47)=3.871, p<0.05, ɳ²p=0.114. That is overall, smaller spatial-temporal scores were observed in the congruent than the incongruent condition (MD=1.32, SE=0.64, p<0.05), and in the spatial component 2 compared to spatial component 1 (MD=9.95, SE=2.72, p<0.05) and 6 (MD=9.00, SE=2.56, p<0.05).

A significant effect was found also for the interaction between congruency and spatial, F(1.74,52.19)=6.741, p<0.01, ɳ²p=0.183. More specifically, a significant difference between congruent and incongruent condition was observed in the following temporal components: ST1, F(1,30)=5.513, p<0.05, ɳ²p=0.155; ST4, F(1,30)=7.820, p<0.01, ɳ²p=0.207; ST5, F(1,30)=9.168, p<0.01, ɳ²p=0.234; ST7, F(1,30)=9.03, p<0.01, ɳ²p=0.231.

In addition, a significant 2-way interaction between the factors spatial and temporal components, F(5.95,178.54)=16.201, p<0.001, ɳ²p=0.351. Significant comparisons are reported in Table S1.

**Figure S1.** Topographic maps of the spatial components. Electrodes whose loadings are greater than average are marked in red.


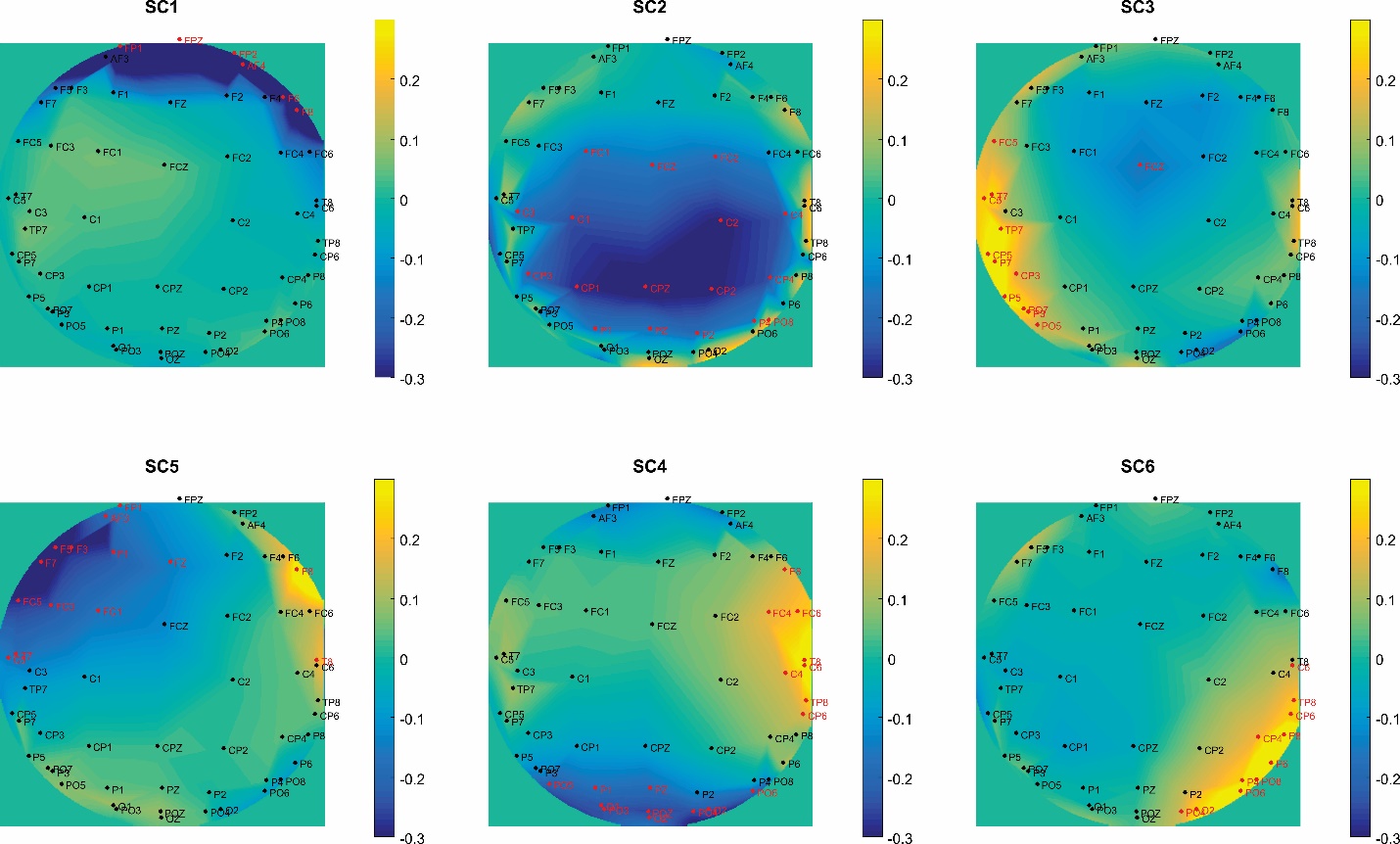


**Figure 2.** Temporal components.


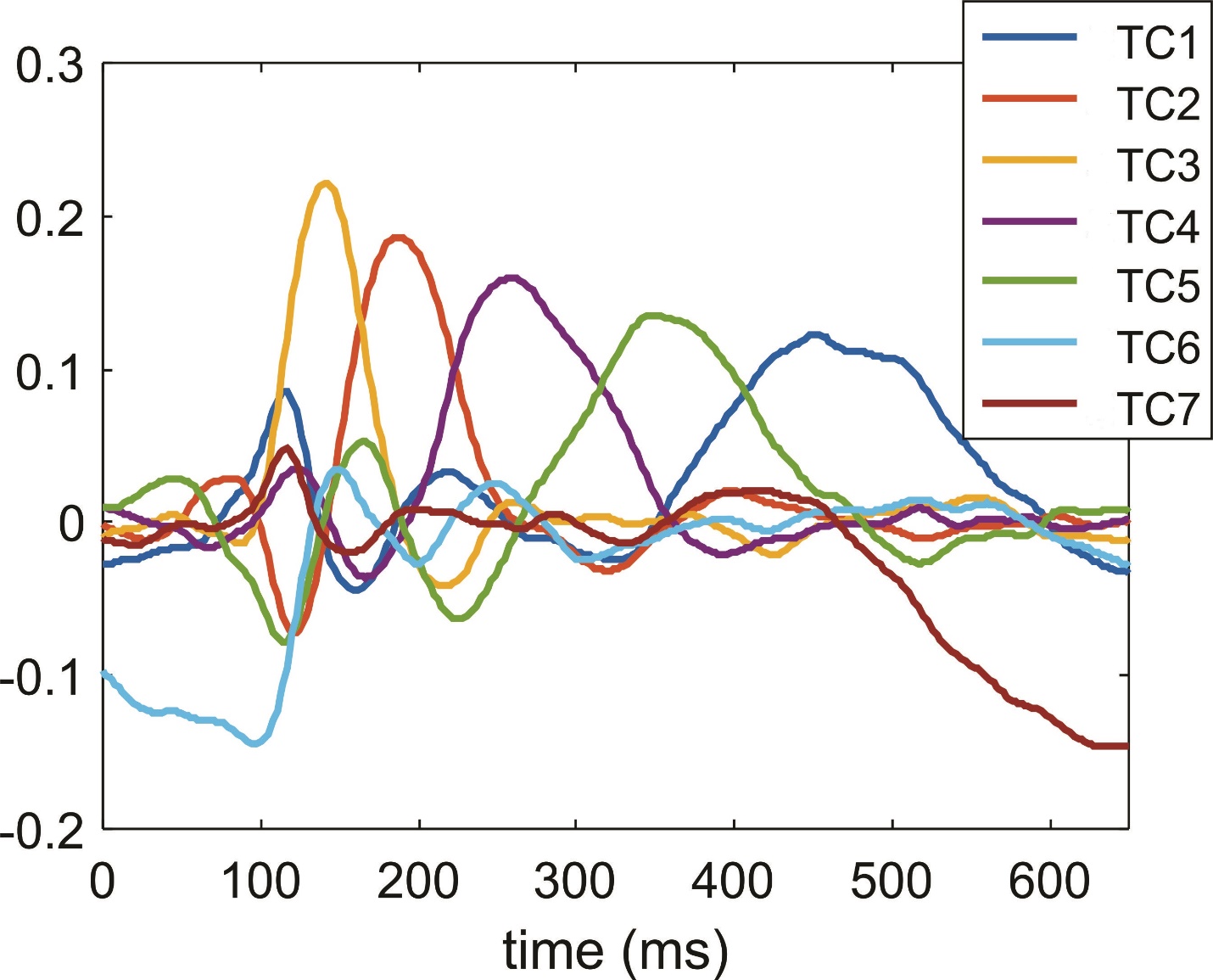


**Table S1.** Significant comparisons between temporal components for each spatial component in the interaction between factors spatial and temporal.

**References**

Dien, J. (2012). Applying Principal Components Analysis to Event-Related Potentials: A Tutorial. *Dev. Neuropsychol.* 37, 497–517.

Dien, J., Spencer, K. M., and Donchin, E. (2003). Localization of the event-related potential novelty response as defined by principal components analysis. *Brain Res. Cogn. Brain Res.* 17, 637–650.

Kayser, J., and Tenke, C. E. (2006). Consensus on PCA for ERP data, and sensibility of unrestricted solutions. *Clin. Neurophysiol.* 117, 695–707.

Lui, M., and Rosenfeld, J. P. (2008). Detection of deception about multiple, concealed, mock crime items, based on a spatial-temporal analysis of ERP amplitude and scalp distribution. *Psychophysiology* 45, 721–730.
